# Exposure to and opinions towards sex education among adolescent students in Mumbai: A cross-sectional survey

**DOI:** 10.1186/1471-2458-11-805

**Published:** 2011-10-14

**Authors:** Tami Benzaken, Ashutosh H Palep, Paramjit S Gill

**Affiliations:** 1 Primary Care Clinical Sciences, University of Birmingham, UK; 2 Ashujay Pharma Pvt.Ltd., Dr.Palep's Medical Research Foundation, Mumbai, India

## Abstract

**Background:**

The aim of this study was to determine students' exposure to sex education and identify students' perceptions of accessibility to sexual health advice and their preferences in implementing sex education.

**Methods:**

A cross-sectional study was carried out in junior colleges in Mumbai in 2010. The self-administered questionnaire investigated male and female students' (aged 15-17) exposure and opinions towards sex education. Data was entered into and analysed using SPSS version 17.0.

**Results:**

The questionnaire was completed by 427 students. Almost 90% of students believed it important to have sex education as part of school curriculum; over 60% reported prior exposure to sex education in school. However, only 45% were satisfied they had good access to advice about contraception and sexual health, particularly, females reported more limited access.

**Conclusions:**

The majority responding indicated a desire for more widespread implementation of school-based sex education, particularly amongst female respondents.

## Background

Sexually transmitted infections (STIs) are among the world's most common diseases; their annual incidence is exceeded only by diarrheal diseases, lower respiratory tract infections and malaria [1]. STIs can result in extensive and devastating physical, social and economic consequences [1,2]. India is in the grip of an HIV epidemic, with an estimated 2.5 million citizens living with HIV [3], and has an annual incidence of STIs estimated at 5% [4].

Adolescents are disproportionally burdened by threats to their sexual health [5,6]. The largest proportion of STIs occur in youth [1,7], with up to 1 in 20 adolescents developing a new STI yearly [1]. In India 31% of existing HIV cases are accounted for by young adults (aged 15-29), despite comprising less than 25% of the population [8]. Whilst there are numerous factors increasing adolescents' vulnerability to poor sexual health outcomes, including physiology [7], economic dependence [8], societal norms and gender imbalances [9]; the lack of access to accurate and comprehensive information regarding sexual health is a key contributor [8,10].

Sex education programmes have proven, 'a cornerstone in reducing adolescent sexual risk behaviours and promoting sexual health' [10]. A review by Kirby et al. evaluated the impact of 83 sex education programmes, demonstrating that sex education programmes have a significant positive impact on young people's risky sexual behaviours [11]. Education programmes were distinguished as being of particular importance to adolescents, regardless of nation or community setting [1] and have been shown to be particularly effective in reducing reported risky sexual behaviours in school going adolescents in developing countries [12]. Furthermore, European countries that have a long history of school-based sex education, such as Switzerland and the Netherlands, demonstrate a correlating trend of low teenage birth rates and low rates of STIs in adolescents [13,14]. However, it is important to acknowledge that other societal, familial, cultural and health service factors exist which may influence rates of teenage pregnancy and STIs in these countries.

In India, sex-related issues are often taboo subjects for discussion [15]. This taboo can be seen to extend into the education system. The Adolescence Education Programme, is a key initiative instigated by the National AIDS Control Organization (NACO) and the Ministry of Human Resource Development in 2002-03. It provides co-curricular (complementing but not part of the regular curriculum) adolescence education in grades 9-12 (ages 14-18). Up until 2007 it had been implemented in almost 150,000 schools across the country [8]. However, since 2007 this sex education programme has been banned in six states including Maharashtra and Karnataka [15,16], where HIV prevalence is highest in India [17]. Given that the few countries that have successfully decreased national HIV prevalence have achieved these gains predominantly by encouraging safer sexual behaviours in adolescents [18], the banning of this programme is of some concern.

Studies have highlighted Indian adolescents' misconceptions in their knowledge of contraception use and STIs [15,19-21]. The Indian National Family Health Survey 2005-06 reported that only 36% of male youths and 20% of females had a comprehensive knowledge of HIV/AIDS [22]. Gupta et al. found that less than one-fifth of participants were aware of STIs other than HIV and only 19.8% of students were aware of at least one method of contraception [19]. The vast gap in adolescents' knowledge of sexual and reproductive health are reflected in their behaviours, with less than 10% of sexually active adolescents in India using contraception [23].

The high prevalence of STIs in India [3,4] is particularly concerning considering the degree to which Indian adolescents contribute to the high figure [8]. Indian adolescents continue to be ignorant of safe sex practices which play a significant part in the current situation [15,19-21]. This is of great concern in view of the current dearth of sex education programmes in India. Consequently, this study aimed to determine students' exposure to sex education and to assess their views on the importance of school-based sex education. It further aimed to identify students' perceptions of accessibility to contraception and sexual health advice and their preferences in implementing sex education.

## Methods

Maharashtra, of which Mumbai is the capital, has one of the highest rates of HIV in India [24]. Moreover, the state's government refused to take part in The Adolescence Education Programme, one federal government initiative to introduce sex education in schools [15,16]. A cross-sectional survey was carried out in five co-educational, state junior colleges in Mumbai, in January and February 2010.

The colleges participating in this study were chosen due to being some of the largest, well-recognised, state junior colleges in Mumbai, distributed in different areas of the city to represent participants from all over the city, consequently they were not randomly sampled.

A self-administered questionnaire (Additional File 1) was developed and piloted for the purpose of this study, with some questions adapted from previously utilised questionnaires [20,25]. The questionnaire, comprising mostly multiple choice questions, was in English as students in the participating colleges are predominantly taught in English. The questionnaire consisted of two sections. The first collected data on students' demographics. The second consisted of questions measuring students' exposure to sex education, their views on accessibility of contraception and sexual health advice and their preferences in implementing sex education.

The questionnaire and an envelope were distributed by the researcher to all students present. Students were selected on the basis of the subject studied (i.e. arts, commerce or science) to ensure a fair representation of students from all subjects. The classes were not organised by sex or academic ability but by subject studied. Classes of the chosen subjects in each college were selected based on convenience sampling for the participating colleges. A total of 5 different classes were included in this study. Prior to distribution of questionnaires an information statement was read to students detailing the aims of the study and how students were selected. A printed copy of this statement was provided to all students. Students were given the opportunity to ask questions about the study. In order to encourage honest responses to the questionnaire, which was of a sensitive nature, students were ensured anonymity and were free to refuse to complete the questionnaire or any parts of it. Responses were returned in sealed envelopes to ensure anonymity and conceal participation. Students who did not wish to participate were asked to return the unanswered questionnaire in a sealed envelope.

All data was entered using SPSS version 17.0 [26]. A sample of 400 male and female students was needed to enable estimation of students' exposure to school-based sex education, with 5% precision and 95% confidence intervals [27]. Frequencies were assessed for each question to determine students' exposure and opinions towards sex education.

### Ethical considerations

Ethical approval was received from the Population Sciences and Humanities Internal Ethics Review Committee, University of Birmingham. Permission to conduct the survey was sought from college principals, and consent forms were signed by appropriate representatives of the junior college in accordance with the college's requirements. (In India principals have the authority to allow cross-sectional surveys to be conducted without parental consent) [20]. Implied consent was assumed from students who completed the questionnaire, or parts of it.

## Results

The study sample comprised of 427 participants (median age 16, range 15-20, 31% male), their demographics characteristics are included in table 1. The majority of students were Hindu (71.7%; n = 306). Among others 13.1% (n = 56) were Muslim, 7% (n = 30) Jain, 3.5% (n = 15) Buddhist and 2.1% (n = 9) Christian. The remainder (n = 10) reported their religion as Sikh, Zoroastrian, Sindhi, Dawoodi Bohra or having no religious affiliation. There was a 100% response rate with the majority of participants completing the questionnaire fully, and 5.4% (n = 23) only partially completing the questionnaire.

**Table 1:** Participant demographics

Characteristic		Total % (n = 427)	Year 11 (n = 410)	Year 12 (n = 17)
Gender		Male: 31% (132)	Male: 29% (119)	Male: 76.5% (13)

		Female: 69% (295)	Female: 71% (291)	Female: 23.5% (4)

Age (years)	15	3.7% (16)	3.9% (16)	-
	16	62.5% (267)	65.1% (267)	-
	17	28.6% (122)	27.1% (111)	64.7% (11)
	18	3.7% (16)	2.4% (10)	35.3% (6)
	19	0.2% (1)	0.2% (1)	-
	20	0.7% (3)	0.7% (3)	-

The majority of students stated 'school' as the most important source of their knowledge regarding contraception and sexual health (Table 2). However, whilst school was the most common source of information for females (72.2%; n = 213. p < 0.001), males reported 'friends' as being the source where the majority of their knowledge comes from (50%; n = 66). Friends were the second most prominent source of knowledge for all students, followed by media sources such as television/radio, magazines/books, internet and cinema. Furthermore, a significantly higher proportion of females reported obtaining information from their parents (27.1%, n = 80) and other family members (23.4%, n = 69, compared to males (8.3%, n = 110 of males, p < 0.001. 9.8%, n = 13, p = 0.01 respectively).

**Table 2:** Sources used by students to gain knowledge of contraception and sexual health

Source of information	Total using source (n = 426)	Males using source (n = 132)	Females using source (n = 294)
**No knowledge**	17 (4%)	7 (5.3%)	10 (3.4%)
**Parent/guardian**	91 (21.3%)	11 (8.3%)	80 (27.1%)
**Other family member**	82 (19.2%)	13 (9.8%)	69 (23.4%)
**School**	277 (64.9%)	64 (48.5%)	213 (72.2%)
**Friend**	221 (51.8%)	66 (50%)	155 (52.5%)
**Doctor**	78 (18.3%)	28 (21.2%)	50 (16.9%)
**Magazines/books**	153 (35.8%)	47 (35.6%)	106 (35.9%)
**TV/radio**	181 (42.4%)	60 (45.5%)	121 (41%)
**Internet**	123 (28.8%)	62 (47%)	61 (20.7%)
**Cinema**	113 (26.5%)	29 (22%)	84 (28.5%)
**Poster/leaflet**	42 (9.8%)	11 (8.3%)	31 (10.5%)
**Other**	6 (1.4%)	5 (3.8%)	1 (0.3%)

(* Multiple responses were noted for single participants.)

Almost two thirds of students (61.6%; n = 263) had previously had sex education lessons at school. Thirty one percent (n = 133) of students had never had sex education at school and 7.3% (n = 31) of students did not remember. Of the students that had prior exposure to sex education the majority of the exposure was in school years 9 and 10 (ages 14-15; 29.3%; n = 125 and 31.9%; n = 136 respectively).

Most of the participants (86.7%; n = 370) believed it important to have sex education as part of the school curriculum, 4.2% (n = 18) did not think it was important and 8.7% (n = 37) were undecided. Almost all of the students who had prior sex education at school (91.6%) thought it was important to have sex education as part of school curriculum.

Forty five percent (n = 192) of students felt they had good access to advice they needed regarding contraception and sexual health. Almost a fifth (17.8%; n = 76) of students did not feel they had good access to advice and 35.4% (n = 151) did not know. However, when comparing the number of males and females who had good access to such advice, 57.6% males (n = 76) and only 39.3% (n = 116), p = 0.002, of females felt they had good access to advice about contraception and sexual health. Out of participants who only partially completed the questionnaire (5.4%), this was the question most commonly unanswered (35%).

Of the students that felt they had insufficient access to advice or did not know if they had good access, the most common reasons attributed were 'not knowing where to go' (30.4%; n = 130) and 'embarrassment' (27.2%; n = 116). However, female participants were almost twice as likely to associate the lack of access to advice with embarrassment compared to males (31.5%; n = 93, compared to 17.4%; n = 23, respectively, p = 0.001). Other reasons that prohibited participants' access were 'fear of parents' (12.4%; n = 53) and 'fear of being told off' (11.2%; n = 48).

Over 60% of participants stated 'doctor' as their preferred source for advice about contraception and sexual health (Table 3). The second most popular choice was 'friend' followed by 'parent/guardian'. Students' favoured format for how they would like to receive sex education was via lectures from trained professionals unrelated to the school (56.2%; n = 240). Approximately half of students wanted to learn from books (50.6%; n = 216). Over a third (36.5%; n = 156) of students wanted the information from lectures given by their school teacher. However, a higher percentage of females wanted lectures from their school teachers (39.7%; n = 117) compared to males (29.5%; 39. p = 0.038).

**Table 3:** Students' preferred point of reference for advice about sexual health

Point of reference for advice	Total (n = 424)	Males (n = 131)	Females (n = 293)
**I don't want advice**	27 (6.3%)	15 (11.4%)	12 (4.1%)
**Doctor**	270 (63.2%)	88 (66.7%)	182 (61.7%)
**Friend**	155 (36.3%)	42 (31.8%)	113 (38.3%)
**Family planning clinic**	102 (23.9%)	33 (25%)	69 (23.4%)
**Parent/guardian**	106 (24.8%)	12 (9.1%)	94 (31.9%)
**Other family member**	45 (10.5%)	6 (4.5%)	39 (13.2%)
**School teacher**	101 (23.7%)	21 (15.9%)	80 (27.1%)
**School nurse**	23 (5.4%)	6 (4.5%)	17 (5.8%)
**Nurse at doctor's surgery**	40 (9.4%)	8 (6.1%)	32 (10.8%)
**Other**	8 (1.9%)	0	8 (2.7%)

## Discussion

This study is one of the few to explore Indian students' preferences in methods of implementing sex education and the barriers preventing them from accessing advice about contraception and sexual health.

### Exposure to sex education

The findings of this study reported an exposure level of 61.6% of students to sex education lessons at school. This figure is significantly higher than previous literature suggests. McManus et al.'s study in Delhi, conducted in 2007 amongst females of a similar age group, found 47% of students had attended classes about STIs, HIV/AIDS or safe sex [20]. The relatively high level of exposure is unexpected in light of the state's government's objection to the Adolescence Education Programme [16].

Despite the moderately high exposure of participants to school-based sex education, the proportion of students who believe sex education to be an important part of the school curriculum (86.7%) greatly exceeds those who receive this education. This finding is strongly supported by previous studies which have found that the majority of students and teachers desire formal, school-based sex education [20,21]. This study suggests that approximately two thirds of students' knowledge regarding contraception and sexual health comes from the school environment. This is contradictory to previous research which suggests that the main sources of information available to adolescents in India about HIV/AIDS, other STIs and safer sex were friends and the media [20]. Whilst friends and the media were the second and third most common sources of knowledge in this study (51.8% and 42.4% respectively), school was the prevailing source.

Given that the vast majority of participating students desire sex education, combined with the fact that for most students schools are the primary source of their knowledge on contraception and sexual health, banning school based sex education may have negative impacts on students' knowledge and ultimately their health. Arguably, there is a need to respond to the collective's desire for sex education. This is further emphasised when considering the high levels of STIs in India, combined with the evidence that the few countries that have successfully decreased national HIV prevalence have achieved this predominantly by encouraging safer sexual behaviours in adolescents [18].

### Implementing sex education

The findings of this study indicate that an overwhelming majority of students' preferred source of advice regarding contraception and sexual health was doctors (63.2%). This supports students' responses that lectures from trained professionals unrelated to the school were the preferred format of delivery of sex education (56.2%). This conflicts with prior literature which suggests that adolescents prefer to consult a relative or friend on reproductive health matters, rather than a health care provider [28]. Students' choice of doctor may suggest that the anonymity and confidentiality afforded by a health care practitioner would enable them to more fully explore this sensitive topic. However, this would not explain why students' choices of consultation with other health care professionals were amongst the least popular (Table 3). The high status attributed to doctors could additionally explain students' choice; students may have felt a higher degree of confidence in consulting a highly ranked professional. Nonetheless, further research is necessary to fully explore this area.

The second most popular response to where students would like to go for advice about contraception and sexual health was friends (36.3%). This may suggest that there is a place for peer-led sex education in schools in India. Whilst peer-led sex education has not been associated with significant improvements in knowledge or behaviour when compared to adult-taught sex education [29], it has been shown to have a greater effect in changing students' attitudes [30].

### Social norms

India's society can be considered a conservative one, where sex-related issues might constitute a taboo for discussion [15]. In spite of this there were no non-responders in this study.

However, the results of this study exhibit other societal norms, in particular that of gender differences, which extend to the norms for sexual behaviour (e.g. premarital sex for women is prohibited but is usually condoned or even encouraged for men) [31]. Hindin et al. reported that 32% of men engage in pre-marital sex, compared to just 6% of women in Delhi [32]. The current study illustrates that female adolescents are more limited in accessing advice about contraception and sexual health, with 57.6% of males stating they have good access to advice they need compared to only 39.3% of females. Female participants were almost twice as likely to associate the lack of access to advice with embarrassment compared to males. Furthermore, a significantly higher proportion of girls (72.2%) receive their knowledge about contraception and sexual health from school compared to boys (48.5%). The fact that adolescent girls are twice as susceptible to getting an STI compared to boys [33] and that they are more dependent on schools for information about sexual health suggests the effect of banning such education may have a differential impact on males and females with significant consequences for female sexual and reproductive health.

This study found that a significantly higher proportion of females obtain information from their parents and other family members compared to male students. This may further demonstrate the societal norm of gender differences. Women are traditionally associated as being the home maker, illustrating that female students might be more at ease seeking information from a family member. Another possibility is that as females are more limited in their access to advice regarding sexual health they have little choice but to seek such information from family members. This further highlights the need for parents to be sensitized to discuss issues pertaining to sexual health, especially with their daughters, as over a quarter of female respondents sought information from their parents.

Moreover, in a country where almost 50% of women are married by 18 and 1 in 6 adolescents are pregnant by the age of 19 [22], it is not merely sex education lessons concerning STIs and contraception that are important to be delivered to female adolescents, but also lessons in reproductive health and family planning.

### Limitations

A major strength of this study is the high response rate (100%) achieved. However, there are a number of limitations such as social desirability bias, as the questionnaire included questions of a sensitive nature. Subjects may have answered some questions in a manner they felt to be socially appropriate or appropriate to the beliefs of the visiting Western researcher. It is impossible to determine in what direction such a bias would have operated but every attempt was made to reassure participants of anonymity and to conceal participation, therefore minimising the bias. Furthermore, responder bias may have occurred as interviews took place in a school environment and students may have, consequently, over-emphasised the importance of knowledge obtained in schools.

A further limitation of this study is the lack of information available on what form of sex education students had previously received; was it one lecture as part of a science lesson or a series of lectures as part of a sex education programme? Consequently, whilst it was possible to establish the prevalence of students' exposure to sex education in schools, it was not possible to determine the extent of sex education received.

This study has explored an important issue given the robust evidence which suggests that curriculum-based sex education programmes are beneficial in preventing HIV, STIs and early pregnancy in adolescents [5]. However, it is important to note that implementing sex education as a preventative measure in schools in India is limited by school attendance rates. Less than 40% of adolescents in India attend secondary school [34]. Consequently, the poorest sectors, who are most at risk of STIs and teenage pregnancy [35], are not represented in this study and would not benefit from school-based sex education.

Additionally, it is important to note that this study defined students' education regarding STIs and contraception as being the main component when referring to sex education. However, sex education is a much wider concept than this and future research would be beneficial exploring differing aspects of sex education such as puberty and family welfare.

## Conclusions

The high levels of HIV and other STIs in India, combined with adolescents' vulnerability, present a challenge in STI prevention. It has been shown that, 'school-based sex education improves awareness of risk and knowledge of risk reduction strategies, increases self-effectiveness and intention to practice safer sex, and delays rather than hastens the onset of sexual activity' [12]. This study has found that despite the relatively high levels of exposure of students to sex education in schools, demand still exceeds supply. Almost 90% of students wanted school-based sex education, which is further supported in the literature [20,21]. Evidently, it is important for culturally sensitive, school-based sex education to be widely implemented.

This study has further shown that Indian female adolescents are particularly limited in their access to information regarding contraception and sexual health. With females more at risk of developing STIs and directly impacted by teenage pregnancy, further research is necessary to explore how to overcome the barriers preventing females from accessing advice they need.

Whilst school-based sex education programmes are an important tool in STI prevention they are not a standalone method. With over 60% of adolescents not attending secondary education [34] attempts to inform young people in India need to be more widespread and further work is needed to explore ways of accessing other sectors of society. Nonetheless, school-based sex education programmes provide an effective means in capturing a large cohort of adolescents, and as this study suggests, is strongly desired by a majority of students in India.

## Competing interests

The authors declare that they have no competing interests.

## Authors' contributions

TB and PSG conceived the study; all three authors developed the protocol; TB undertook data collection and statistical analysis with input from PSG. All authors read and approved the final manuscript.

## Pre-publication history

The pre-publication history for this paper can be accessed here:

http://www.biomedcentral.com/1471-2458/11/805/prepub

## Supplementary Material

Additional file 1 **Questionnaire**. This is a copy of the questionnaire administered to the children. Click here for file
