# Supplementary material for: Exposure to and opinions towards sex education among adolescent students in Mumbai: A cross-sectional survey
Source: BMC Public Health. 2011 Oct 14;11:805. doi: 10.1186/1471-2458-11-805 (PMC3236074; doi:10.1186/1471-2458-11-805)
Supplement: Additional file 1 — Questionnaire. This is a copy of the questionnaire administered to the children. [file 1471-2458-11-805-S1.DOCX]

**Appendix 1: Questionnaire**


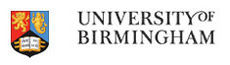


**Exposure and opinions towards sex education:**

**A Survey of adolescent students in Mumbai, India**

**2010**

This questionnaire is anonymous and all your responses will be confidential. Your honest response to questions is important. If you do not wish to answer one of the questions please leave it blank.

Most questions require you to tick () the answer you think is appropriate.

**Section A**

This section asks you about yourself

A.1 Are you male or female? (Please tick correct box)

| **Male** |  |
| --- | --- |
| **Female** |  |

A.2 Age (in years): ______

A.3 What class/grade/form are you in at junior college? ­­______

A.4 What is your religion?

| **Hindu** |  |
| --- | --- |
| **Sikh** |  |
| **Muslin** |  |
| **Christian** |  |
| **Buddhist** |  |
| **Jain** |  |
| **No religious affiliation** |  |

Other (please specify): ____________________________

**Section B**

This section asks you about where you receive information about contraception (birth control) and sexually transmitted infections (STIs). STIs are infections that a person can get by having sex.

B.1 Where did most of your knowledge about contraception (birth control) and sexual health come from? (You may tick as many boxes as necessary)

| **I have no knowledge of contraception or sexual health** |  |
| --- | --- |
| **Parents/guardian** |  |
| **Other family member (brother, sister, cousin…)** |  |
| **School** |  |
| **Friend** |  |
| **Doctor** |  |
| **Magazines/books** |  |
| **TV/radio** |  |
| **Internet** |  |
| **Cinema** |  |
| **Poster/leaflet** |  |

Other (please specify):________________________

B.2 Do you feel you have good access to the advice you need?

| **Yes** |  |
| --- | --- |
| **No** |  |
| **Don’t know** |  |

B.3 What stops you from getting the advice and help you need, if applicable?

(You may tick as many boxes as necessary)

| **Embarrassment** |  |
| --- | --- |
| **Don’t know where to go** |  |
| **Fear of being told off** |  |
| **Fear of parents/guardian finding out** |  |

Other (please specify):________________________

B.4 Have you ever had formal, sex education lessons at school?

| **Yes** |  |
| --- | --- |
| **No** |  |
| **Can’t remember** |  |

If yes, in what year were you when you received the lessons? ______________________

B.5 Do you think it is important to have sexual health education as part of your school curriculum?

| **Yes** |  |
| --- | --- |
| **No** |  |
| **Don’t know** |  |

B.6 Who/where would you like to be able to go for advice about contraception and sexual health?

| **I don’t want any advice about contraception and sexual health** |  |
| --- | --- |
| **Doctor** |  |
| **Friend** |  |
| **Family planning Clinic** |  |
| **Parents/guardian** |  |
| **Other family member** |  |
| **I don’t want any advice** |  |
| **School teacher** |  |
| **School nurse** |  |
| **Nurse at doctor surgery** |  |

Other (please specify):_______________________

B.7 If you were to receive formal sexual education in which format would you most like to receive the information? (You may tick as many boxes as necessary)

| **Lectures from your current teachers** |  |
| --- | --- |
| **Lectures from trained professionals not related to the school** |  |
| **Videos** |  |
| **Books** |  |
| **Leaflets** |  |

Other (please specify):____________________________________________

**Thank you for your help in completing this questionnaire.**
